# Persistent symptoms in euthyroid Hashimoto’s thyroiditis: current hypotheses and emerging management strategies

**DOI:** 10.3389/fendo.2025.1627787

**Published:** 2025-07-18

**Authors:** Hui Zhang, Wenting Tong, Weiyong Zeng, Hongyan Luo, Licai Zhang, Jiasheng Feng, Yang Xiao, Gankun Wang

**Affiliations:** ^1^ Department of Urology, Dongguan Hospital affiliated to Guangzhou University of Chinese Medicine, Dongguan, China; ^2^ Endocrinology and Medical Sexology (ENDOSEX), Department of Experimental Medicine, University of Rome Tor Vergata, Rome, Italy; ^3^ Department of Pharmacy, Gannan Healthcare Vocational College, Ganzhou, Jiangxi, China; ^4^ The Ninth Clinical Medical College, Guangzhou University of Chinese Medicine, Guangzhou, China; ^5^ Department of Nephrology, Dongguan Hospital affiliated to Guangzhou University of Chinese Medicine, Dongguan, China; ^6^ Department of Thyroid Surgery, Dongguan Hospital affiliated to Guangzhou University of Chinese Medicine, Dongguan, China

**Keywords:** Hashimoto’s thyreoiditis, biochemical euthroidism, therapy, autoimmunity modulation, symptoms

## Abstract

A substantial proportion of patients with Hashimoto’s thyroiditis (HT) continue to experience persistent symptoms despite achieving biochemical euthyroidism, either with or without levothyroxine (LT4) replacement therapy. Several pathophysiological mechanisms have been proposed to explain this clinical phenomenon, including a reduced free triiodothyronine to free thyroxine (FT3/FT4) ratio and persistently elevated thyroid autoantibody titers. Escalation of LT4 monotherapy is generally discouraged due to an unfavourable risk–benefit profile. In contrast, combined LT4 and liothyronine (LT3) therapy may offer symptomatic improvement in selected patients, though robust criteria for selection remain undefined. More recently, total thyroidectomy has been explored as a therapeutic option in patients with refractory symptoms, demonstrating sustained improvements in health-related quality of life compared to medical therapy. However, this surgical approach must be balanced against potential perioperative risks and complications. Adjunctive therapies, including selenium and vitamin D supplementation, have shown modest benefit. In parallel, emerging evidence has highlighted the potential of traditional Chinese medicine (TCM)—notably, herbal medicine and acupuncture—as a complementary strategy for symptom relief, although further high-quality studies are warranted. This review synthesizes current insights into the mechanisms underlying residual symptoms in HT and critically evaluates contemporary and emerging therapeutic approaches aimed at improving patient-reported outcomes and long-term disease management

## 1 Introduction

Hypothyroidism is a common endocrine disorder characterized by insufficient thyroid hormone production, estimated to affect a range of 4.8–25.8% of women and 0.9–7.9% of men in the general population, with a variable prevalence due to different definitions used and the population studied (1–3). The most frequent cause of hypothyroidism in iodine-sufficient countries is Hashimoto’s thyroiditis (HT), an autoimmune condition characterized by lymphocytic infiltration of the thyroid gland that leads to thyroid gland fibrosis and atrophy. Initially described in Japan over a century ago as a pronounced lymphoid goitre predominantly affecting women, nowadays, not only is HT recognized as the most common cause of hypothyroidism but also as the most common autoimmune and endocrine disease (4). It encompasses several pathologic variants (classic form, fibrous variant, juvenile form, etc.), all of which are characterized by the infiltration of hematopoietic mononuclear cells, mainly lymphocytes, into the thyroid interstitium. While patients can present as euthyroid or even hyperthyroid (a process known as hashitoxicosis), most HT forms ultimately lead to hypothyroidism. Diagnosis relies on detecting circulating antibodies against thyroid antigens, primarily anti-thyroperoxidase (TPO-Ab or anti-TPO) and anti-thyroglobulin (Tg-Ab or anti-Tg), and observing reduced echogenicity on thyroid ultrasonography in patients exhibiting appropriate clinical features. Treatment remains symptomatic, focusing on administering levothyroxine (LT4) supplementation to correct hypothyroidism as needed (5).

Recent research suggests that approximately 5-10% of, patients with Hashimoto’s thyroiditis may experience persisting symptoms such as. Fatigue, weight gain, cold intolerance, constipation and, depression (6),despite achieving biochemical euthyroidism, as defined by normal levels of thyroid stimulating hormone (TSH) and free-thyroxine (FT4) (7) Several hypotheses have been proposed to explain this phenomenon (**Table 1**).

**Table 1:** Strategies available for the management of euthyroid patients with Hashimoto thyroiditis with persistent symptoms and their related mechanisms.

Mechanism to address	Therapy	Impact on treatment	Evidence quality	Clinical relevance
Individual variability in optimal TSH threshold	Increasing LT4 therapy in order to maintain TSH levels in the lower part of the appropriate reference range	It may give modest improvements in resting energy expenditure, but at increased cardiovascular and osteoporotic risks	Moderate (randomized controlled trials)	Low (not recommended by guidelines)
Immunological/ Inflammatory	Thyroidectomy Selenium, vitamin D Herbal medicine Acupuncture	Significant clinical improvement, normalization of fatigue and anti-TPO, but with risks of postsurgical complications May reduce anti-TPO titers and modulate Inflammation, but clinical benefit is uncertain May reduce anti-TPO titers, exert antioxidant action and modulate inflammation, but clinical benefit is uncertain May reduce anti-TPO titers, modulate Inflammation and improve quality of life, but long-term clinical benefit is uncertain	Moderate (randomized controlled trials, reviews) Low–moderate (randomized controlled trials, reviews) Low–moderate (reviews, small randomized controlled trials) Low–moderate (reviews, small randomized controlled trials)	High for patients with high anti-TPO titers or persistent symptoms, but typically reserved for patients with compressive symptoms, cytological suspicion of malignancy, or coexisting nodular disease Moderate; consider supplementation in deficient patients Low; efficacy is promising but more and better-quality studies are needed Low; efficacy is promising but more and better-quality studies are needed
Impaired FT4 conversion to FT3	Combination of LT4 and LT3 therapy	Combination therapy may raise the FT3 to FT4 ratio and help some patients	Low–moderate (reviews, small randomized controlled trials)	Moderate; consider in symptomatic patients with low FT3
Genetic (e.g. polymorphisms in *DIO2* gene)	Combination of LT4 and LT3 therapy	Combination therapy may raise the FT3 to FT4 ratio and help some patients with impaired deiodinase or thyroid hormone transporter polymorphisms	Low (hypothesis, limited data)	Low evidence available for the efficacy of genotyping in predicting response with combination therapy

LT4, levothyroxine; LT3, liothyronine; anti-TPO, anti-thyroperoxidase.

First, while TSH remains the cornerstone for monitoring thyroid function, its role as a sole marker of tissue-level euthyroidism has been questioned. Although TSH shows a logarithmic amplification in response to minor changes in FT4 and free triiodothyronine (FT3), it reflects pituitary thyroid hormone sensitivity but may not accurately represent peripheral thyroid hormone activity (8). TSH operates within a tightly regulated feedback loop involving the hypothalamic-pituitary-thyroid (HPT) axis, but this feedback is non-linear and affected by a large individual variability in set points (9). Such inter-individual differences in TSH-FT4-FT3 relationships are shaped by genetic, metabolic, and environmental factors, meaning that a “normal” TSH may reflect a relative, rather than absolute, thyroid hormone status. In clinical practice, this suggests that TSH does not reliably indicate tissue-level euthyroidism in the context of LT4 therapy, and it can translate into persistent symptoms of hypothyroidism despite apparently adequate replacement (10). Secondly, TSH predominantly reflects pituitary sensitivity to circulating T4, rather than tissue-specific availability or utilisation of the active hormone T3. This dissociation becomes especially relevant in patients receiving LT4 monotherapy. While TSH may normalise with LT4 treatment, peripheral tissues may still experience inadequate T3 supply due to a lack of endogenous T3 production and variable deiodinase activity (11, 12). Deiodinase activities are specifically regulated in tissues, and their regulation may have important consequences for the peripheral effects of thyroid hormone and for the setpoints of feedback regulation (13). Indeed, impaired conversion of T4 to the biologically active T3 may underlie persistent symptoms in some patients. There are several polymorphisms in the genes of deiodinases and in thyroid hormone transporters that may influence tissue T3 availability. For instance, individuals carrying polymorphisms in the deiodinase type 2 gene (DIO2), such as the common Thr92Ala variant, have an altered enzymatic activity and impaired T3 generation at the tissue level, potentially explaining the limited clinical response to LT4 monotherapy in these patients (14–16). In individuals carrying this polymorphism, while the biochemical thyroid profile may appear normal, cellular hypothyroidism might persist, contributing to subjective complaints.

Third, the autoimmune nature of Hashimoto’s thyroiditis may itself contribute significantly to persistent symptoms, even when thyroid hormone levels are within the reference ranges (7, 17, 18). In a large systematic review of studies, Groenewegen et al. provide compelling evidence that chronic inflammation and immune dysregulation associated with thyroid autoimmunity may directly affect patient well-being, independent of biochemical thyroid status (7). Specifically, individuals with positive TPO-Ab report a higher prevalence of symptoms such as fatigue, cognitive dysfunction, irritability, and depressive mood—even when their TSH and FT4 levels are normal. The underlying mechanisms may involve sustained low-grade inflammation driven by ongoing immune activation. TPO-Ab positivity, often used as a marker of thyroid autoimmunity, has been associated with elevated pro-inflammatory cytokines, including interleukin-6 (IL-6) and tumour necrosis factor-alpha (TNF-α) (19, 20), which can have systemic effects beyond the thyroid gland (21, 22). These cytokines may influence central nervous system function through neuroinflammatory pathways and have been implicated in the pathophysiology of fatigue, mood disturbances, and altered pain perception. However, although numerous studies have demonstrated an association between thyroid autoimmunity and persistent symptoms in biochemically euthyroid patients with Hashimoto’s thyroiditis, the underlying causative mechanisms remain poorly understood, and current evidence is largely based on observational data rather than interventional or mechanistic studies. While further research is needed to clarify the causal pathways, the potential role of autoimmunity as a driver of persistent symptoms should not be overlooked in clinical practice.

**Figure 1:**
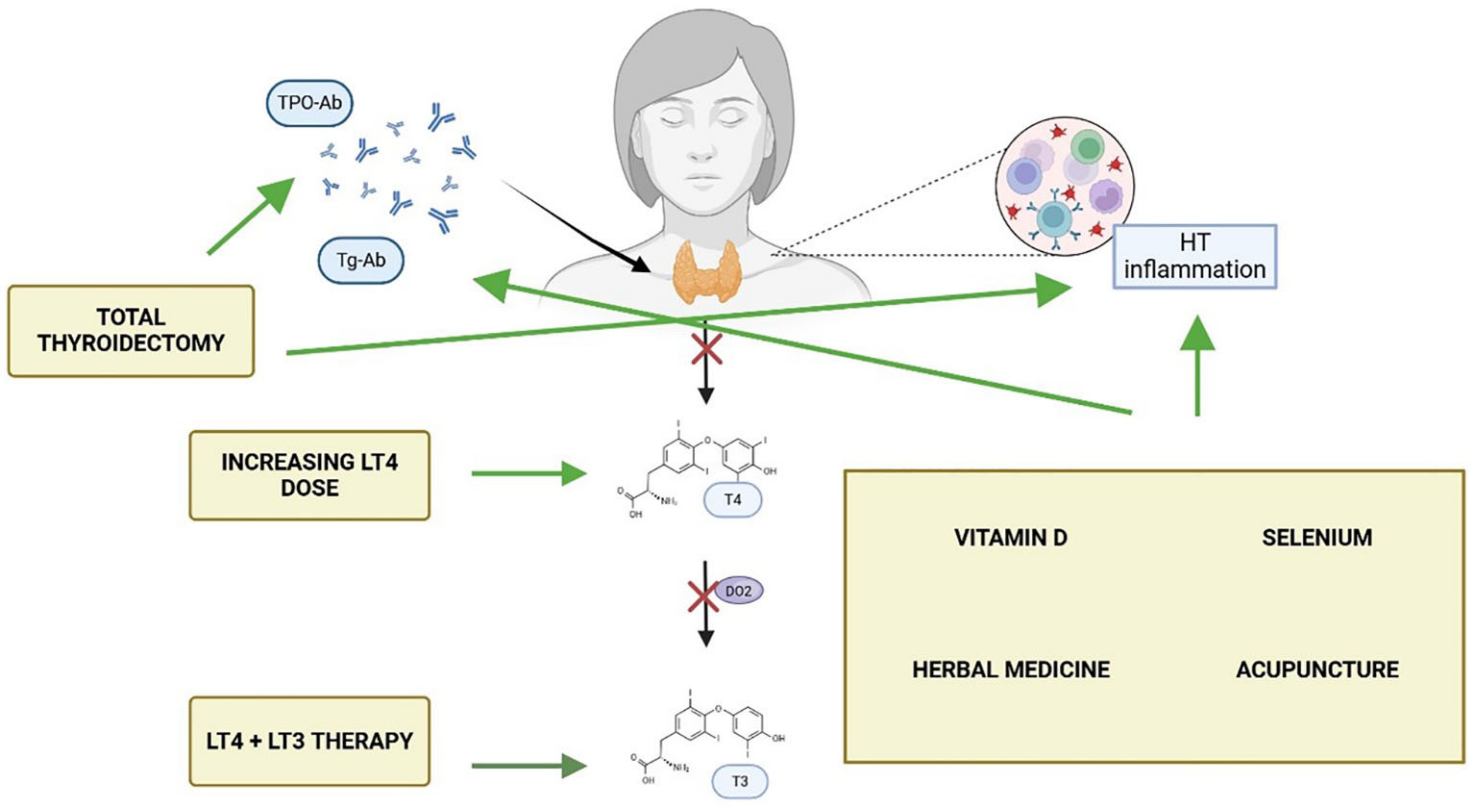
Currently available strategies, and their mechanisms of action, for patients with Hashimoto thyroiditis (HT) with persisting symptoms despite normal thyroid hormone levels. Made with BioRender.com.

In light of these mechanisms, the prevailing assumption that biochemical euthyroidism equates to clinical remission in HT should be challenged, highlighting the need to broaden the clinical framework beyond hormone levels to include markers of immune activity, inflammation, and patient-reported outcomes. These elements may better reflect the lived experience of patients with autoimmune thyroid disease. Moreover, despite increasing awareness, there is currently no consensus on how to manage persistent symptoms in biochemically euthyroid individuals with HT. This narrative review aims to synthesise the current evidence on the most frequently proposed management strategies for this patient subset (**Figure 1**), critically examining their pathophysiological underpinnings and clinical efficacy. In doing so, the review seeks to offer a structured overview of an under-recognised yet clinically relevant challenge in endocrinology.

## 2 Providing increasing doses of LT4 therapy

The optimal treatment for hypothyroidism remains a subject of debate, with the etiology and severity of hypothyroidism and the amount of residual functional thyroid tissue affecting the LT4 dosage (23, 24) Moreover, in light of the complexities surrounding thyroid hormone homeostasis, a one-size-fits-all approach to TSH targets may be inappropriate, particularly given the interindividual variability in TSH set-points and peripheral hormone metabolism (8, 23, 25). This personalised framework is especially relevant across diverse populations, where physiological and pathological conditions influence both thyroid hormone requirements and appropriate TSH targets. For instance, in pregnancy, the increased demand for thyroid hormones necessitates lower TSH thresholds, with current guidelines recommending levels below 2.5 mU/L in the first trimester and below 3.0 mU/L thereafter (26, 27). In contrast, elderly individuals often exhibit a physiological shift in the set-point of the HPT axis with aging, transitioning toward higher TSH levels with age; in these cases, mildly elevated TSH values (up to 6–7.5 mU/L) may not reflect true hypothyroidism and do not necessarily require intervention, particularly in the absence of symptoms. Similarly, in patients with coronary artery disease or other cardiovascular comorbidities, a conservative approach with higher TSH targets within the normal range is typically preferred to avoid precipitating cardiac complications. In obesity, where leptin-driven hypothalamic stimulation can lead to modest TSH elevations despite normal FT4 levels and negative autoantibodies, treatment decisions should carefully weigh the risk of overtreatment. Children and adolescents, on the other hand, require age-specific TSH and FT4 targets to ensure proper growth and neurodevelopment (5, 8). Taken together, these considerations foster the importance of individualizing LT4 replacement therapy, in order to align it not only with biochemical targets but also with the clinical context and physiological nuances of each patient (28). However, while standard therapy for HT aims to normalize serum TSH levels within the reference range, some patients continue to experience symptoms despite achieving this target. This phenomenon has prompted growing interest in the mechanisms that may underlie non-responsiveness to LT4 monotherapy. One important mechanism is the impaired peripheral conversion of T4 to the biologically active T3, a process largely dependent on the activity of deiodinase enzymes. Genetic polymorphisms in the *DIO2* gene—particularly the common Thr92Ala variant—have been associated with altered enzymatic function and reduced intracellular T3 availability in key tissues, despite normal serum TSH and FT4 levels (14–16, 29). This impaired conversion may lead to tissue-specific hypothyroidism not captured by standard thyroid function tests. Additionally, genetic variants in the phosphodiesterase 8B (*PDE8B*) gene, which encodes a high-affinity cAMP-specific phosphodiesterase, as genetic modulators of TSH levels (30, 31), and in the TSH receptor (32) may also influence the setpoint of the HPT axis, potentially contributing to diminished cellular responsiveness to circulating thyroid hormones. Beyond these molecular factors, extrathyroidal influences such as chronic inflammation, comorbid autoimmune disorders, and psychological stress can modulate thyroid hormone action at the tissue level, further complicating symptom resolution (6, 7). One proposed strategy to address persistent symptoms has been to adjust LT4 dosing in order to maintain TSH levels in the lower part of the reference range (e.g., 0.4–2.0 mU/L), based on the hypothesis that doing so may more closely approximate the physiological set-point and potentially improve clinical outcomes. Indeed, lowering TSH to this range has been associated with modest improvements in resting energy expenditure and reductions in fat mass in hypothyroid patients (33). However, no consistent benefits have been demonstrated in terms of broader metabolic parameters or QoL (33). This hypothesis was directly tested in a double-blind, randomized controlled trial (RCT) by Walsh et al. (34), which evaluated whether targeting TSH in the lower part of the reference range would alleviate persistent symptoms and enhance QoL in patients with primary hypothyroidism. The study, involving middle-aged and older patients, found no significant improvement in well-being and cognitive function with a lower TSH target compared to standard replacement therapy aimed at mid- to upper-normal TSH levels. These findings suggest that simply adjusting LT4 doses to push TSH toward the lower end of the reference range does not address the underlying causes of persistent symptoms and may expose patients to increased risks. Indeed, higher LT4 doses are associated with a greater risk of adverse outcomes, including atrial fibrillation, osteoporosis, and fractures (35–37). For these reasons, current guidelines do not recommend attempting to improve symptoms by lowering TSH targets beyond the standard reference range (5). Notably, no studies to date have specifically tested this hypothesis in patients with Hashimoto’s thyroiditis, limiting the generalisability of existing evidence to this particular population; nevertheless, it is advisable to adhere to current treatment guidelines and to explore alternative, evidence-based approaches that address the underlying mechanisms contributing to persistent symptoms in this subset of patients.

## 3 LT4+LT3 combination therapy

When monitoring the efficacy of LT4 therapy, adequacy is traditionally assessed by TSH and FT4 levels. However, whether LT4 administration is also able to restore FT3 values and whether decrements in FT3 that remain within the reference range are actually of any clinical significance is a matter of controversy (28, 38). For instance, FT3 levels may remain in the low-normal reference range in a significant subset of thyroidectomized patients receiving replacement doses of LT4 (12). To date, most patients on LT4 with normal serum TSH exhibit a decreased serum T3/T4 ratio (39). To explain this phenomenon, many hypotheses have been proposed, includingthe reduced efficiency of peripheral deionisation of exogenous T4 compared to endogenous thyroidal secretion (12); the tendency for TSH to normalize at higher serum T4 levels without sufficient serum T3 restoration (40); and genetic factors, such as the Thr92Ala polymorphism in the *DIO2* gene (rs225014) which is found in 12–36% of the population (15). Notably, this polymorphism reduces deiodinase type 2 enzymatic velocity by about 20% and might explain the lower serum T3 in LT4-treated hypothyroid carriers (14, 29). However, a consensus regarding the clinical significance of reduced T3 and whether this should be corrected is unknown (5, 41).Evidence from clinical trials of LT4+LT3 combination therapy is mixed. A 2022 systematic review and meta-analysis of 18 randomized trials including 883 hypothyroid patients and evaluating outcomes like depression, fatigue, pain, anxiety, and anger, found no statistically significant advantage of combined LT4+LT3 therapy over LT4 monotherapy on any of these psychological endpoints (42). Despite this, combination therapy was more commonly preferred by the patients. Therefore, while the biochemical addition of T3 raises T3/T4 ratios, clinical benefits remain unproven in broad populations, and current guidelines do not support its routine use (5). However, it may be considered in selected patients with persistent symptoms despite adequate LT4 dosing and normal TSH, provided that alternative explanations for their complaints have been excluded (5). The rationale is that combination therapy may more closely replicate the physiological secretion ratio of T4 to T3 (approximately 13:1 by weight), potentially restoring a more physiological FT3/FT4 balance (43). Moreover, while earlier studies suggested that carriers of the Thr92Ala polymorphism in the *DIO2* gene might experience reduced T3 availability and a preference for LT4+LT3 combination therapy (44), more recent evidence has prompted a re-evaluation of the role of genotyping in guiding individualized therapy for hypothyroidism. A large-scale study on the UK Biobank found no significant association between Thr92Ala carriage and symptomatic improvement, FT3 concentrations, or treatment preference, thus questioning its value as a predictive biomarker (45). Hence, so far a conclusive evidence of improved outcomes with combination therapy remains lacking, and the benefits appear limited to specific individuals rather than the broader population of patients with Hashimoto thyroiditis and persisting symptoms (46).

## 4 Surgery

In recent years, several studies have suggested that persistent symptoms in HT patients may be linked to the autoimmune process (47). Serum TPO-Ab may contribute to systemic inflammation through cross-reactivity with other tissues; for instance, elevated levels of Hsp60, structurally similar to Tg and TPO, have been shown to cross-react with both Tg-Ab and TPO-Ab (48). Additionally, TPO-Ab enhance pro-inflammatory cytokine responses to TPO in mononuclear cells (20), contributing to inflammation and extra-thyroidal diseases (49).Ott et al., demonstrated that elevated pre-operative TPO-Ab levels were significantly associated with fatigue, irritability, and reduced QoL in euthyroid patients undergoing surgery for benign goitre (18). While hypothyroidism is managed with thyroid hormone therapy (either LT4 or LT4+LT3), the underlying autoimmune component remains unaddressed. Indeed, research on the effects of LT4 therapy on TPO-Ab in HT patients shows mixed results. Schmidt et al. found that TPO-Ab levels decreased in 92% of patients over 50 months of LT4 treatment, with a mean decrease of 70% after 5 years. However, only 16% of patients achieved negative TPO-Ab levels (47). Romaldini et al. observed a significant decrease in TPO-Ab levels in clinical hypothyroidism patients, but not in subclinical cases (50). These findings have led some researchers to explore whether addressing the autoimmune process more directly could improve patient outcomes. One emerging hypothesis is that thyroidectomy may offer therapeutic benefit in selected patients with Hashimoto’s thyroiditis who continue to experience significant symptoms despite achieving biochemical euthyroidism with LT4 therapy (51). In this context, the abatement of humoral thyroid autoimmunity through total thyroidectomy has been hypothesised to attenuate the ongoing autoimmune response, potentially alleviating systemic symptoms such as fatigue, mood disturbances, and cognitive impairment (52). Indeed, several studies have reported post-operative improvements in quality of life, mental health, and fatigue levels among patients undergoing surgery for persistent, non-compressive symptoms (**Table 2**). These effects appear to be independent of thyroid hormone levels, reinforcing the idea that the inflamed thyroid gland may itself contribute to symptom persistence via immune-mediated mechanisms. Notably, the only RCT available to date by Guldvog et al. showed significant improvements in fatigue and QoL at 6, 12, and 18 months after total thyroidectomy compared to medical therapy alone. These improvements occurred alongside a marked reduction in TPO-Ab titres and were hypothesised to result from immunological downregulation following removal of the antigenic stimulus (**Figure 1**). Indeed, evidence suggests that the clearance of TPO-Ab seems to occur in parallel with other immunologic mediators such as interferon-γ, TNF-α, interleukin-1ß, and interleukin-2, as well as C-reactive protein (54, 55) which might explain the benefit of the complete removal of the antigenic tissue via total thyroidectomy. On the other hand, in a more recent study, the same authors of the prior RCT found that preoperative TPO-Ab levels did not appear to influence the odds ratio to obtain a clinically significant improvement in QoL, which might not fully support the hypothesis of autoimmunity being directly responsible for persistent symptoms in patients with euthyroid HT (56).

**Table 2:** Studies that evaluated postoperative outcomes in patients with Hashimoto thyroiditis with persistent, non compressive symptoms.

Authors and year	Study design	Sample size	Follow-up period	Outcomes	Conclusions	Complications
Hoff et al., 2024 (56)	Prospective, observational study	154	18 months	36-Item Short Form Survey (SF-36); General Health score; anti-TPO antibody titers	Significant clinical improvement; significant anti-TPO reduction	Permanent unilateral recurrent nerve palsy (1.9%), hypoparathyroidism (3.9%)
Serrao-Brown et al., 2024 (86)	Retrospective case-control study	30 (Hashimoto thyroiditis group)	Not specified	SF-36 and Thyroid- Related Patient- Reported Outcome-39 (ThyPRO-39) quality of life scores; anti-TPO, anti-TG	Significant clinical improvement; significant reduction of antibody titers	No complications
Thatipamala et al., 2020 (87)	Retrospective cohort study	19	3-35 months	SF-36 general health score (only postoperative); disease management questionnaire	62.5% general health improvement; 87.5% feeling moderately or extremely happy with their decision to proceed with thyroidectomy; 43.8% improved energy level	Temporary hypoparathyroidism (47,4%); temporary unilateral vocal fold paresis (5,2%)
Guldvog et al., 2019 (53)	Randomized controlled trial	73 (surgical group) vs 74 (control group)	18 months	SF-36 general health subscore; anti-TPO titers; fatigue scores	Significant clinical improvement, normalization of fatigue and anti-TPO; results achieved only in the surgical group	Postsurgical infections (4.1%), longstanding hypocalcemia (4.1%), unilateral recurrent nerve palsy (5.5%)
Zivaljevic V.R., et al, 2015 (88)	Prospective cohort study	27 (Hashimoto thyroiditis group)	6 months	ThyPRO-39 quality of life score	Significant clinical improvement in all domains – except eye symptoms and cognitive impairment	Not specified

TPO, thyroperoxidase; TG, thyroglobulin.

Surgical risks, however, must be weighed carefully in this patient population. A large prospective multicentre study by Thomusch et al. involving over 18,000 patients found that while general complications were rare and comparable between patients undergoing surgery for autoimmune thyroid disease and those with multinodular goitre, the risk of transient (15.3%) and permanent (1.1%) hypoparathyroidism was significantly higher in HT patients than in those undergoing surgery for multinodular goitre (12.9% and 0.9%, respectively) (57). A possible explanation could be the dense inflammatory process that surrounds the thyroid gland in HT, which makes visual identification and preservation of parathyroids more complicated. By contrast, rates of vocal cord palsy—both transient and permanent—were comparable across groups, and surgery for HT did not emerge as an independent risk factor for laryngeal nerve injury. Importantly, the extent of thyroidectomy (i.e. total vs. subtotal) and poor identification or preservation of the parathyroid glands were identified as key contributors to the risk of hypoparathyroidism, highlighting the need for careful surgical planning and intraoperative management to mitigate complications. Hence, from a clinical standpoint, surgery for HT is not a first-line option and is typically reserved for patients with compressive symptoms, cytological suspicion of malignancy, or coexisting nodular disease (51). In the context of persistent symptoms without compressive signs, the decision for thyroidectomy is guided by multidisciplinary evaluation and shared decision-making, where patient preference, symptom burden, and exclusion of non-thyroidal causes are carefully weighed. In this framework, surgery may be considered a valid option only after exhausting conservative medical approaches.

Importantly, a recent cost-effectiveness analysis using a Markov decision model showed that total thyroidectomy may be both more effective and less costly over the long term than continued medical therapy in euthyroid HT patients with disabling symptoms (58). This is due primarily to the substantial indirect costs related to reduced work productivity from HT-associated depression and anxiety—conditions for which patients with HT have markedly elevated odds ratios (3.56 and 2.32, respectively) (21). Despite the upfront cost and risk of surgery, these findings suggest that the long-term burden of inadequately treated autoimmune symptoms may outweigh the risks, positioning surgery as a potentially rational choice in selected patients. Rather than viewing persistent symptoms as inevitable, this growing body of evidence underscores the importance of a proactive and individualised management strategy for HT.

## 5 Supplements

The literature on the clinical management of HT devotes considerable attention to the role of nutritional supplements, particularly selenium and vitamin D, in modulating the autoimmune process and alleviating persistent symptoms (59–61). Selenium, a trace element highly concentrated in the thyroid gland, is essential for the activity of several selenoproteins involved in antioxidant defence and thyroid hormone metabolism; thyrocytes express a large number of selenoproteins that have a wide range of functions, from antioxidant and anti-inflammatory roles to the production of active thyroid hormone, like type I and type II deiodinase (62). Studies have linked selenium deficiency with higher autoantibody titers in patients with HT, suggesting a potential immunomodulatory role (63); nonetheless, selenium supplementation has not been shown to cause any apparent improvements in the clinical course of the disease and the evidence for selenium’s efficacy remains inconclusive due to limited high-quality studies (64).More recently, in a meta-analysis, Wichman et al. found that selenium supplementation significantly reduced TPO-Ab levels in LT4-treated patients after 3, 6, and 12 months, but whether these effects correlate with clinically relevant measures remains to be demonstrated (65). Notably, the authors highlighted significant methodological limitations across the included studies, such as small sample sizes, short follow-up durations, and high heterogeneity in baseline selenium status, dosage regimens, and patient populations. These factors limit the generalisability of findings and preclude firm conclusions regarding the long-term effectiveness of selenium in HT management.

Similarly, vitamin D supplementation has been investigated for its immunoregulatory potential in HT. Low vitamin D levels are consistently observed in patients with autoimmune thyroiditis, and mechanistic studies suggest that vitamin D may help restore immune tolerance by modulating T-cell activity and cytokine profiles (66, 67). In HT, the vitamin D-induced prevention of dendritic cells- dependent T-cell activation and inflammatory cytokines production and the restoration of the anti-inflammatory state through a restoration of the Th17/Treg ratio has been claimed as one of the beneficial mechanisms (68, 69). Some studies have found that vitamin D supplementation in HT patients with vitamin D deficiency led to decreased thyroid autoantibody titers (70, 71) and improved thyroid function. However, the results are not always confirmed (59, 72), and trials often suffer from the same limitations noted in selenium research: small cohorts, brief observation periods, and lack of standardisation in dosing and baseline vitamin D threshold (61). Moreover, very few studies rigorously assess patient-centred outcomes such as fatigue, mood, or quality of life—symptoms that remain central concerns in euthyroid HT.

Other supplements like zinc, vitamin B12, and myo-inositol have also been studied, but their efficacy is less established (60, 61). Although preliminary findings suggest potential benefit in reducing autoantibodies or improving metabolic markers, these results are primarily drawn from small-scale or uncontrolled studies and lack replication in high-quality RCTs.In summary, while the biological rationale for supplement use in HT is compelling, robust clinical evidence remains limited. Most available studies are underpowered, short-term, and heterogeneous in design, making it difficult to draw firm conclusions about long-term efficacy, optimal dosing strategies, or the specific patient subgroups most likely to benefit. Future research should focus on well-designed, adequately powered trials with standardised outcome measures and longer follow-up periods to determine the true value of nutritional supplementation in the management of persistent symptoms in euthyroid HT.

## 6 Herbal medicine

More recently, the interest in the use of herbal medicine for HT has grown, particularly within the context of traditional Chinese medicine (TCM). A recent meta-analysis of 16 trials investigated 12 Chinese herbal formulas, three Chinese patent medicines, and one single herbal medicine highlighting both immunological and symptomatic outcomes in HT patients (73). The Chinese Yiqi Huayu Recipe (*Astragalus membranaceus* (Fisch.) Bge., *Codonopsis pilosula* (Franch.) Nannf., *Angelica archangelica* L., *Atractylodes macrocephala* Koidz., *Ligusticum chuanxiong* Hort., *Paeonia lactiflora* Pall., *Curcuma longa* L., *Poriacocos* (Schw.) Wolf., *Citrus × aurantium* L., *Pinellia ternata* (Thunb.) Makino, *Fritillaria thunbergii* Miq.) 10 gr twice/day resulted in the best reduction of both TPOAb and TGAb compared to placebo, but changes in clinical symptoms were not evaluated. Conversely, the Qi-invigorating, phlegm-resolving, and blood-activating formula, containing *Astragalus mongholicus* Bunge, *Panax ginseng* C.A.Mey., *Angelica archangelica* L., *Curcuma longa* L., *Pinellia ternata* (Thunb.) Makino, *Fritillaria thunbergii* Miq., Oyster, Carapax Trionycis, taken, three times per day was the most effective in symptoms reduction. The findings may suggest that these remedies may be used as potential therapeutic targets for HT treatment; however, the mechanism by which these herbs decrease thyroid antibody levels remains unclear, requiring further modern pharmacology to explain their therapeutic effects. Emerging pharmacological evidence indicates that Chinese herbal formulations exert multifaceted immunomodulatory effects relevant to HT (74–76). Specifically, many active compounds appear to inhibit the TLR4/NF-κB signalling pathway, thereby downregulating the expression of pro-inflammatory cytokines such as IL-6, TNF-α, and IL-1β (77, 78). Others act via SIRT1/Nrf2/NF-κB pathway, protecting thyrocytes from apoptosis and limiting the release of thyroid autoantigens (79). Additionally, some herbal compounds promote regulatory T-cell responses while inhibiting Th17 differentiation, a key imbalance in HT pathogenesis (75). Antioxidant effects, such as the enhancement of superoxide dismutase and glutathione peroxidase, also contribute to thyrocyte preservation (80). Recent findings further suggest the possibility of improving HT by regulating intestinal microbiota (81), with a possible role of herbal treatments in modulating the gut–immune axis. Beyond these mechanistic insights, a large ethnopharmacological survey of 104 studies identified *Dioscorea nipponicae*, *Prunellae* sp*ica*, *Astragali*, and *Cordyceps sinensis* as key herbs consistently associated with thyroid antibody reduction and anti-inflammatory activity (82). Nevertheless, the current body of evidence is limited by small sample sizes, short follow-up durations, heterogeneous herbal formulations, and a general lack of standardisation in dose and quality control. Moreover, many studies fail to assess clinically relevant endpoints such as fatigue, cognitive dysfunction, or quality of life. As such, while Chinese herbal medicine offers a promising avenue for HT symptom management, further high-quality, long-term clinical trials are needed to establish its efficacy, safety, and optimal use in routine care (59, 73, 82).

## 7 Acupuncture

Recent studies suggest acupuncture may offer complementary benefits for the management of HT, particularly for patients with persistent symptoms despite achieving biochemical euthyroidism. A 2023 systematic review and meta-analysis that evaluated a total of 1020 patients participating in 14 RCTs found that acupuncture significantly regulated TPOAb and TGAb and hormones (FT3, FT4, TSH) compared to levothyroxine alone (83). These findings support a potential immunomodulatory effect of acupuncture; however, the clinical significance remains uncertain. Indeed, when evaluating the clinical symptoms as shown by the scores of the Hospital Anxiety Scale (HADS-A) and Hospital Depression Scale (HADS-D), there was no significant difference between acupuncture and levothyroxine sodium tablets. Moreover, a substantial methodological heterogeneity, including inconsistent protocols, lack of blinding, and unclear randomisation, limits the robustness of the conclusions.

**Figure 2:**
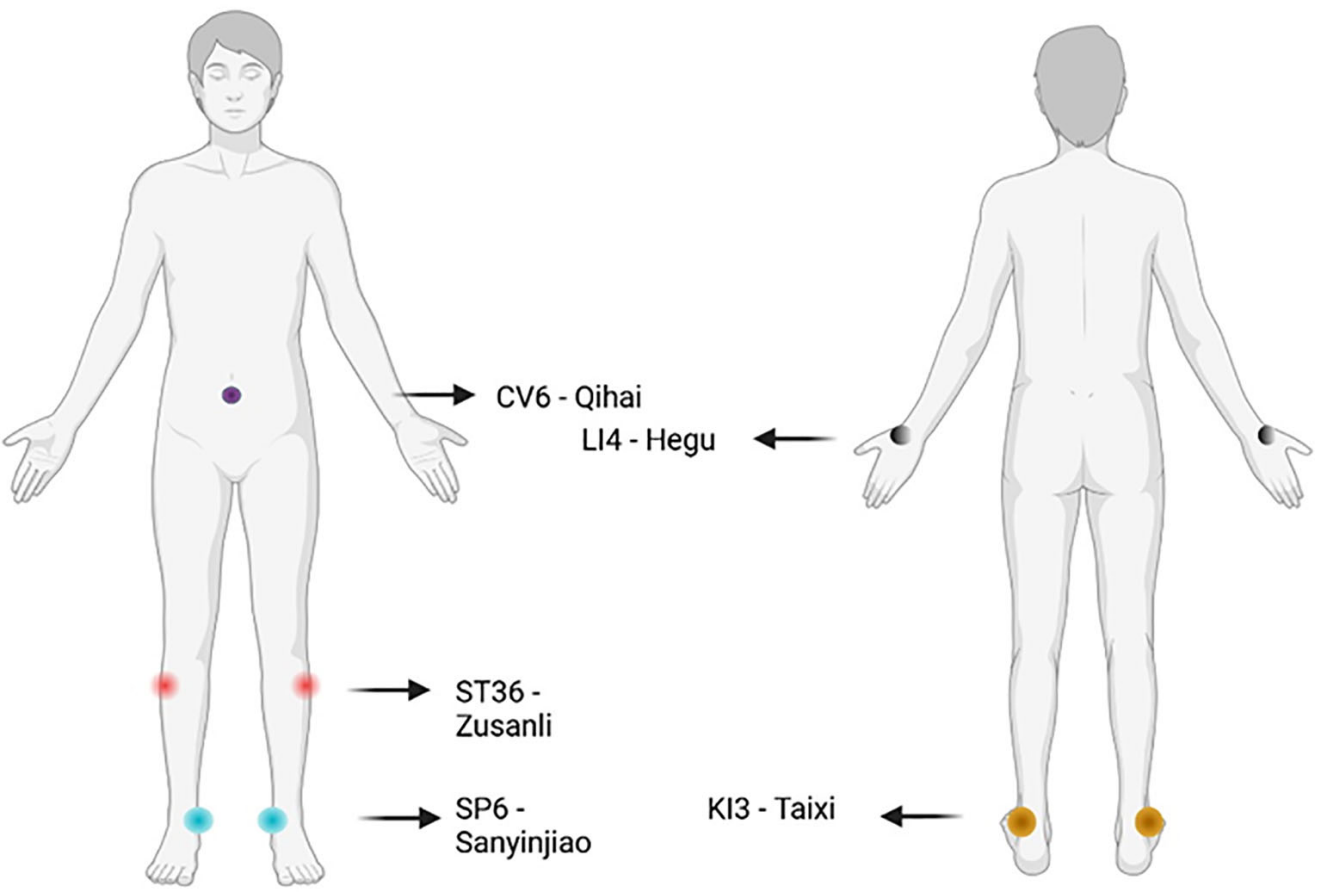
Most commonly employed acupoints for Hashimoto thyroiditis used in clinical trials. Made with BioRender.com.

Beyond clinical data, acupuncture’s rationale is grounded in TCM theory, which conceptualises HT as a disorder of “spleen and kidney yang deficiency” or “liver qi stagnation leading to phlegm obstruction.” These imbalances are thought to disrupt the body’s internal harmony and immune defence. Acupuncture aims to restore systemic equilibrium by stimulating specific acupoints and meridians to reinforce qi, dissipate phlegm, and unblock meridian stagnation (84). Commonly used points include ST36 (*Zusanli*) and SP6 (*Sanyinjiao*) to tonify the spleen and kidney, KI3 (*Taixi*) to nourish essence and yin, and CV6 (*Qihai*) for qi regulation (83) (**Figure 2**).

An exploratory RCT employing Hand Yangming Meridian Penetrating Acupuncture, targeting points along the large intestine meridian such as LI4 (Hegu), found improvements in quality of life and mental health domains of the SF-36 and HADS-A, alongside reductions in TgAb titres (85). However, due to the small sample size (27 in the acupuncture group, 25 in the waiting list group) and the lack of correction for multiple testing, results were not statistically robust. Still, the study remains noteworthy as the first RCT of its kind evaluating acupuncture specifically for HT.

Nevertheless, significant methodological limitations persist across the current literature. Most trials are in Chinese and limited by small sample sizes, short follow-up periods, and heterogeneous protocols, including variation in acupoint selection, treatment duration, adjunctive techniques (e.g., electroacupuncture, moxibustion), and underlying TCM diagnoses. Additionally, few studies use standardised symptom scales or evaluate long-term clinical endpoints such as fatigue, cognitive function, or sustained quality of life improvements. Blinding and allocation concealment are often inadequately reported, increasing the risk of bias.

In conclusion, while preliminary results of acupuncture are encouraging, especially in terms of antibody reduction and select QoL improvements, rigorous, larger-scale, and theory-driven RCTs are needed to determine its definitive role in the management of euthyroid HT with persistent symptoms.

## 8 Conclusion

Managing patients with Hashimoto thyroiditis who continue to experience symptoms despite achieving biochemical euthyroidism remains a significant clinical challenge. While standard LT4 monotherapy effectively restores thyroid hormone levels in most patients, it does not fully address persistent symptoms in a subset of individuals. This review highlights the limitations of conventional approaches and explores alternative strategies, including LT4+LT3 combination therapy, total thyroidectomy, and adjunctive treatments such as supplements, herbal medicine, and acupuncture. Although emerging evidence supports the potential benefits of these interventions, current data remain inconclusive, and individualized treatment approaches are necessary. Future research should focus on identifying biomarkers to guide personalized management and conducting rigorous, long-term comparative studies to determine the most effective therapeutic strategies for this subset of patients.

## References

[1] Garmendia Madariaga A Santos Palacios S Guillén-Grima F Galofré JC. The incidence and prevalence of thyroid dysfunction in Europe: a meta-analysis. J Clin Endocrinol Metab. (2014) 99:923–31. doi: 10.1210/jc.2013-2409, PMID: 24423323

[2] Taylor PN Albrecht D Scholz A Gutierrez-Buey G Lazarus JH Dayan CM. Global epidemiology of hyperthyroidism and hypothyroidism. Nat Rev Endocrinol. (2018) 14:301–16. doi: 10.1038/nrendo.2018.18, PMID: 29569622

[3] Hu X Chen Y Shen Y Tian R Sheng Y Que H. Global prevalence and epidemiological trends of Hashimoto’s thyroiditis in adults: A systematic review and meta-analysis. Front Public Health. (2022) 10. doi: 10.3389/fpubh.2022.1020709, PMID: 36311599 PMC9608544

[4] Caturegli P De Remigis A Rose NR. Hashimoto thyroiditis: clinical and diagnostic criteria. Autoimmun Rev. (2014) 13:391–7. doi: 10.1016/j.autrev.2014.01.007, PMID: 24434360

[5] Jonklaas J Bianco AC Bauer AJ Burman KD Cappola AR Sawka AM. Guidelines for the treatment of hypothyroidism: prepared by the american thyroid association task force on thyroid hormone replacement. Thyroid. (2014) 24:1670–751. doi: 10.1089/thy.2014.0028, PMID: 25266247 PMC4267409

[6] Saravanan P Chau WF Roberts N Vedhara K Greenwood R Dayan CM. Psychological well-being in patients on ‘adequate’ doses of l-thyroxine: results of a large, controlled community-based questionnaire study. Clin Endocrinol (Oxf). (2002) 57:577–85. doi: 10.1046/j.1365-2265.2002.01654.x, PMID: 12390330

[7] Groenewegen KL Mooij CF van Trotsenburg ASP. Persisting symptoms in patients with Hashimoto’s disease despite normal thyroid hormone levels: Does thyroid autoimmunity play a role? A systematic review. J Transl Autoimmun. (2021) 4:100101. doi: 10.1016/j.jtauto.2021.100101, PMID: 34027377 PMC8122172

[8] Biondi B Wartofsky L. Treatment with thyroid hormone. Endocr Rev. (2014) 35:433–512. doi: 10.1210/er.2013-1083, PMID: 24433025

[9] Andersen S Pedersen KM Bruun NH Laurberg P. Narrow individual variations in serum T(4) and T(3) in normal subjects: a clue to the understanding of subclinical thyroid disease. J Clin Endocrinol Metab. (2002) 87:1068–72. doi: 10.1210/jcem.87.3.8165, PMID: 11889165

[10] Razvi S Bhana S Mrabeti S. Challenges in interpreting thyroid stimulating hormone results in the diagnosis of thyroid dysfunction. J Thyroid Res. (2019) 2019:4106816. doi: 10.1155/2019/4106816, PMID: 31662841 PMC6778876

[11] Jonklaas J Davidson B Bhagat S Soldin SJ. Triiodothyronine levels in athyreotic individuals during levothyroxine therapy. JAMA. (2008) 299:769–77. doi: 10.1001/jama.299.7.769, PMID: 18285588

[12] Gullo D Latina A Frasca F Le Moli Pellegriti G Vigneri R. Levothyroxine monotherapy cannot guarantee euthyroidism in all athyreotic patients. PloS One. (2011) 6:e22552. doi: 10.1371/journal.pone.0022552, PMID: 21829633 PMC3148220

[13] Maia AL Kim BW Huang SA Harney JW Larsen PR. Type 2 iodothyronine deiodinase is the major source of plasma T3 in euthyroid humans. J Clin Invest. (2005) 115:2524–33. doi: 10.1172/JCI25083, PMID: 16127464 PMC1190373

[14] Castagna MG Dentice M Cantara S Ambrosio R Maino F Porcelli T. DIO2 thr92Ala reduces deiodinase-2 activity and serum-T3 levels in thyroid-deficient patients. J Clin Endocrinol Metab. (2017) 102:1623–30. doi: 10.1210/jc.2016-2587, PMID: 28324063

[15] Dora JM MaChado WE Rheinheimer J Crispim D Maia AL. Association of the type 2 deiodinase Thr92Ala polymorphism with type 2 diabetes: case-control study and meta-analysis. Eur J Endocrinol. (2010) 163:427–34. doi: 10.1530/EJE-10-0419, PMID: 20566590

[16] Butler PW Smith SM Linderman JD Brychta RJ Alberobello AT Dubaz OM. The thr92Ala 5′ Type 2 deiodinase gene polymorphism is associated with a delayed triiodothyronine secretion in response to the thyrotropin-releasing hormone–stimulation test: A pharmacogenomic study. Thyroid. (2010) 20:1407–12. doi: 10.1089/thy.2010.0244, PMID: 21054208 PMC2990280

[17] Bazzichi L Rossi A Giuliano T De Feo F Giacomelli C Consensi A. Association between thyroid autoimmunity and fibromyalgic disease severity. Clin Rheumatol. (2007) 26:2115–20. doi: 10.1007/s10067-007-0636-8, PMID: 17487449

[18] Ott J. Hashimoto’s thyroiditis affects symptom load and quality of life unrelated to hypothyroidism: A prospective case–control study in women undergoing thyroidectomy for benign goiter. Thyroid®. (2011) 21:161–7. doi: 10.1089/thy.2010.0191, PMID: 21186954

[19] Ru X Yang M Teng Y Han Y Hu Y Wang J. Association of maternal thyroid peroxidase antibody during pregnancy with placental morphology and inflammatory and oxidative stress responses. Front Endocrinol. (2023) 14. doi: 10.3389/fendo.2023.1182049, PMID: 37810887 PMC10556745

[20] Nielsen CH Brix TH Leslie RGQ Hegedüs L. A role for autoantibodies in enhancement of pro-inflammatory cytokine responses to a self-antigen, thyroid peroxidase. Clin Immunol. (2009) 133:218–27. doi: 10.1016/j.clim.2009.07.014, PMID: 19726232

[21] Siegmann EM Müller HH Luecke C Philipsen A Kornhuber J Grömer TW. Association of depression and anxiety disorders with autoimmune thyroiditis: A systematic review and meta-analysis. JAMA Psychiatry. (2018) 75:577–84. doi: 10.1001/jamapsychiatry.2018.0190, PMID: 29800939 PMC6137529

[22] Kirim S Keskek SÖ Köksal F Haydardedeoglu FE Bozkirli E Toledano Y. Depression in patients with euthyroid chronic autoimmune thyroiditis. Endocr J. (2012) 59:705–8. doi: 10.1507/endocrj.EJ12-0035, PMID: 22673294

[23] Sukumar R Agarwal A Gupta S Mishra A Agarwal G Verma AK. Prediction of LT4 replacement dose to achieve euthyroidism in subjects undergoing total thyroidectomy for benign thyroid disorders. World J Surg. (2010) 34:527–31. doi: 10.1007/s00268-009-0345-3, PMID: 20044749

[24] Gordon MB Gordon MS. Variations in adequate levothyroxine replacement therapy in patients with different causes of hypothyroidism. Endocr Pract. (1999) 5:233–8. doi: 10.4158/EP.5.5.233, PMID: 15251659

[25] Biondi B. The normal TSH reference range: what has changed in the last decade? J Clin Endocrinol Metab. (2013) 98:3584–7. doi: 10.1210/jc.2013-2760, PMID: 24014812

[26] De Groot L Abalovich M Alexander EK Amino N Barbour L Cobin RH. Management of thyroid dysfunction during pregnancy and postpartum: an Endocrine Society clinical practice guideline. J Clin Endocrinol Metab. (2012) 97:2543–65. doi: 10.1210/jc.2011-2803, PMID: 22869843

[27] Stagnaro-Green A Abalovich M Alexander E Azizi F Mestman J Negro R. Guidelines of the American Thyroid Association for the diagnosis and management of thyroid disease during pregnancy and postpartum. Thyroid. (2011) 21:1081–125. doi: 10.1089/thy.2011.0087, PMID: 21787128 PMC3472679

[28] Ettleson MD Bianco AC. Individualized therapy for hypothyroidism: is T4 enough for everyone? J Clin Endocrinol Metab. (2020) 105:e3090–104. doi: 10.1210/clinem/dgaa430, PMID: 32614450 PMC7382053

[29] Gereben B McAninch EA Ribeiro MO Bianco AC. Scope and limitations of iodothyronine deiodinases in hypothyroidism. Nat Rev Endocrinol. (2015) 11:642–52. doi: 10.1038/nrendo.2015.155, PMID: 26416219 PMC5003781

[30] Arnaud-Lopez L Usala G Ceresini G Mitchell BD Pilia MG Piras MG. Phosphodiesterase 8B gene variants are associated with serum TSH levels and thyroid function. Am J Hum Genet. (2008) 82:1270–80. doi: 10.1016/j.ajhg.2008.04.019, PMID: 18514160 PMC2427267

[31] Taylor PN Panicker V Sayers A Shields B Iqbal A Bremner AP. A meta-analysis of the associations between common variation in the PDE8B gene and thyroid hormone parameters, including assessment of longitudinal stability of associations over time and effect of thyroid hormone replacement. Eur J Endocrinol. (2011) 164:773–80. doi: 10.1530/EJE-10-0938, PMID: 21317282 PMC3080745

[32] Medici M van der Deure WM Verbiest M Vermeulen SH Hansen PS Kiemeney LA. A large-scale association analysis of 68 thyroid hormone pathway genes with serum TSH and FT4 levels. Eur J Endocrinol. (2011) 164:781–8. doi: 10.1530/EJE-10-1130, PMID: 21367965

[33] Al-Adsani H Hoffer LJ Silva JE. Resting energy expenditure is sensitive to small dose changes in patients on chronic thyroid hormone replacement. J Clin Endocrinol Metab. (1997) 82:1118–25. doi: 10.1210/jc.82.4.1118, PMID: 9100583

[34] Walsh JP Ward LC Burke V Bhagat CI Shiels L Henley D. Small changes in thyroxine dosage do not produce measurable changes in hypothyroid symptoms, well-being, or quality of life: results of a double-blind, randomized clinical trial. J Clin Endocrinol Metab. (2006) 91:2624–30. doi: 10.1210/jc.2006-0099, PMID: 16670161

[35] Sawin CT. Low serum thyrotropin concentrations as a risk factor for atrial fibrillation in older persons. N Engl J Med. (1994) 331:1249–52. doi: 10.1056/NEJM199411103311901, PMID: 7935681

[36] Collet TH Gussekloo J Bauer DC den Elzen WP Cappola AR Balmer P. Subclinical hyperthyroidism and the risk of coronary heart disease and mortality. Arch Intern Med. (2012) 172:799–809. doi: 10.1001/archinternmed.2012.402, PMID: 22529182 PMC3872478

[37] Faber J Galløe AM. Changes in bone mass during prolonged subclinical hyperthyroidism due to L-thyroxine treatment: a meta-analysis. Eur J Endocrinol. (1994) 130:350–6. doi: 10.1530/eje.0.1300350, PMID: 8162163

[38] Cooper DS. Thyroxine monotherapy after thyroidectomy: coming full circle. JAMA. (2008) 299:817–9. doi: 10.1001/jama.299.7.817, PMID: 18285594

[39] Peterson SJ McAninch EA Bianco AC. Is a normal TSH synonymous with “Euthyroidism” in levothyroxine monotherapy? J Clin Endocrinol Metab. (2016) 101:4964–73. doi: 10.1210/jc.2016-2660, PMID: 27700539 PMC6287526

[40] De Castro JPW Fonseca TL Ueta CB McAninch EA Abdalla S Wittmann G. Differences in hypothalamic type 2 deiodinase ubiquitination explain localized sensitivity to thyroxine. J Clin Invest. (2015) 125:769–81. doi: 10.1172/JCI77588, PMID: 25555216 PMC4319436

[41] Abdalla SM Bianco AC. Defending plasma T3 is a biological priority. Clin Endocrinol (Oxf). (2014) 81:633–41. doi: 10.1111/cen.2014.81.issue-5, PMID: 25040645 PMC4699302

[42] Lan H. Combined T4 + T3 therapy versus T4 monotherapy effect on psychological health in hypothyroidism: A systematic review and meta-analysis. Clin Endocrinol (Oxf). (2022) 97:13–25. doi: 10.1111/cen.14742, PMID: 35445422

[43] Wiersinga WM Duntas L Fadeyev V Nygaard B Vanderpump MPJ. ETA guidelines: the use of L-T4 + L-T3 in the treatment of hypothyroidism. Eur Thyroid J. (2012) 1:55–71. doi: 10.1159/000339444, PMID: 24782999 PMC3821467

[44] Carlé A Faber J Steffensen R Laurberg P Nygaard B. Hypothyroid patients encoding combined MCT10 and DIO2 gene polymorphisms may prefer L-T3 + L-T4 combination treatment - data using a blind, randomized, clinical study. Eur Thyroid J. (2017) 6:143–51. doi: 10.1159/000469709, PMID: 28785541 PMC5527224

[45] Jensen CZ Isaksen JL Ahlberg G Olesen MS Nygaard B Ellervik C. Association of DIO2 and MCT10 polymorphisms with persistent symptoms in LT4-treated patients in the UK biobank. J Clin Endocrinol Metab. (2024) 109:e613–22. doi: 10.1210/clinem/dgad556, PMID: 37740545

[46] Vargas-Uricoechea H Wartofsky L. LT4/LT3 combination therapy vs. Monotherapy with LT4 for persistent symptoms of hypothyroidism: A systematic review. Int J Mol Sci. (2024) 25:9218. doi: 10.3390/ijms25179218, PMID: 39273168 PMC11395006

[47] Schmidt M. Long-term follow-up of antithyroid peroxidase antibodies in patients with chronic autoimmune thyroiditis (Hashimoto’s thyroiditis) treated with levothyroxine. Thyroid. (2008) 18:755–60. doi: 10.1089/thy.2008.0008, PMID: 18631004

[48] Gammazza AM Rizzo M Citarrella R Rappa F Campanella C Bucchieri F. Elevated blood Hsp60, its structural similarities and cross-reactivity with thyroid molecules, and its presence on the plasma membrane of oncocytes point to the chaperonin as an immunopathogenic factor in Hashimoto’s thyroiditis. Cell Stress Chaperones. (2014) 19:343–53. doi: 10.1007/s12192-013-0460-9, PMID: 24057177 PMC3982029

[49] Fröhlich E Wahl R. Thyroid autoimmunity: role of anti-thyroid antibodies in thyroid and extra-thyroidal diseases. Front Immunol. (2017) 8:521. doi: 10.3389/fimmu.2017.00521, PMID: 28536577 PMC5422478

[50] Romaldini JH Biancalana MM Figueiredo DI Farah CS Mathias PC. Effect of L-thyroxine administration on antithyroid antibody levels, lipid profile, and thyroid volume in patients with Hashimoto’s thyroiditis. Thyroid. (1996) 6:183–8., PMID: 8837324 10.1089/thy.1996.6.183

[51] McManus C Luo J Sippel R Chen H. Should patients with symptomatic hashimoto’s thyroiditis pursue surgery? J Surg Res. (2011) 170:52–5. doi: 10.1016/j.jss.2011.01.037, PMID: 21435660 PMC3136540

[52] Chiovato L Latrofa F Braverman LE Pacini F Capezzone M Masserini L. Disappearance of humoral thyroid autoimmunity after complete removal of thyroid antigens. Ann Intern Med. (2003) 139:346–51. doi: 10.7326/0003-4819-139-5_Part_1-200309020-00010, PMID: 12965943

[53] Guldvog I Reitsma LC Johnsen L Lauzike A Gibbs C Carlsen E. Thyroidectomy versus medical management for euthyroid patients with hashimoto disease and persisting symptoms: A randomized trial. Ann Intern Med. (2019) 170:453–64. doi: 10.7326/M18-0284, PMID: 30856652

[54] Karanikas G Schuetz M Wahl K Paul M Kontur S Pietschmann P. Relation of anti-TPO autoantibody titre and T-lymphocyte cytokine production patterns in Hashimoto’s thyroiditis. Clin Endocrinol (Oxf). (2005) 63:191–6. doi: 10.1111/j.1365-2265.2005.02324.x, PMID: 16060913

[55] Krysiak R Okopien B. The effect of levothyroxine and selenomethionine on lymphocyte and monocyte cytokine release in women with Hashimoto’s thyroiditis. J Clin Endocrinol Metab. (2011) 96:2206–15. doi: 10.1210/jc.2010-2986, PMID: 21508145

[56] Hoff G Bernklev T Johnsen L Reitsma L Sina D Lauzike A. Thyroidectomy for euthyroid patients with hashimoto disease and persistent symptoms: an observational, postrandomization study. J Thyroid Res. (2024) 2024:5518720. doi: 10.1155/2024/5518720, PMID: 38606313 PMC11008973

[57] Thomusch O Sekulla C Billmann F Seifert G Dralle H Lorenz K. Risk profile analysis and complications after surgery for autoimmune thyroid disease. Br J Surg. (2018) 105:677–85. doi: 10.1002/bjs.10770, PMID: 29579336

[58] Memeh K Ruhle B Vaghaiwalla T Kaplan E Keutgen X Angelos P. Thyroidectomy for euthyroid patients with Hashimoto thyroiditis and persisting symptoms: A cost-effectiveness analysis. Surgery. (2021) 169:7–13. doi: 10.1016/j.surg.2020.03.028, PMID: 32460999

[59] Luo J Zhou L Sun A Min Y Lin Y Han L. Clinical comparative efficacy and therapeutic strategies for the Hashimoto’s thyroiditis: A systematic review and network meta-analysis. Heliyon. (2024) 10:e35114. doi: 10.1016/j.heliyon.2024.e35114, PMID: 39247354 PMC11379579

[60] Michalska S Makuch R Gała K Lenard P Kucharski A Pilarski K. The role of micronutrient supplementation in the management of hashimoto’s thyroiditis: A review of current evidence and potential mechanisms of action. Qual Sport. (2024) 20:53265–5. doi: 10.12775/QS.2024.20.53265

[61] Peng B Wang W Gu Q Wang P Teng W Shan Z. Effects of different supplements on Hashimoto’s thyroiditis: a systematic review and network meta-analysis. Front Endocrinol (Lausanne). (2024) 15:1445878. doi: 10.3389/fendo.2024.1445878, PMID: 39698034 PMC11652148

[62] Winther KH Rayman MP Bonnema SJ Hegedüs L. Selenium in thyroid disorders - essential knowledge for clinicians. Nat Rev Endocrinol. (2020) 16:165–76. doi: 10.1038/s41574-019-0311-6, PMID: 32001830

[63] Rostami R Nourooz-Zadeh S Mohammadi A Khalkhali HR Ferns G Nourooz-Zadeh J. Serum selenium status and its interrelationship with serum biomarkers of thyroid function and antioxidant defense in hashimoto’s thyroiditis. Antioxidants. (2020) 9:1070. doi: 10.3390/antiox9111070, PMID: 33142736 PMC7692168

[64] van Zuuren EJ Albusta AY Fedorowicz Z Carter B Pijl H. Selenium supplementation for Hashimoto’s thyroiditis. Cochrane Database Syst Rev. (2013) 2013:CD010223. doi: 10.1002/14651858.CD010223.pub2, PMID: 23744563 PMC9862303

[65] Wichman J Winther KH Bonnema SJ Hegedüs L. Selenium supplementation significantly reduces thyroid autoantibody levels in patients with chronic autoimmune thyroiditis: A systematic review and meta-analysis. Thyroid. (2016) 26:1681–92. doi: 10.1089/thy.2016.0256, PMID: 27702392

[66] Durá-Travé T Gallinas-Victoriano F. Autoimmune thyroiditis and vitamin D. Int J Mol Sci. (2024) 25:3154. doi: 10.3390/ijms25063154, PMID: 38542128 PMC10969999

[67] Barragan M Good M Kolls JK. Regulation of dendritic cell function by vitamin D. Nutrients. (2015) 7:8127–51. doi: 10.3390/nu7095383, PMID: 26402698 PMC4586578

[68] Rydzewska M Jaromin M Pasierowska IE. Stożek, K. & Bossowski, A. Role of the T and B lymphocytes in pathogenesis of autoimmune thyroid diseases. Thyroid Res. (2018) 11:2. doi: 10.1186/s13044-018-0046-9, PMID: 29449887 PMC5812228

[69] Soda M Priante C Pesce C De Maio G Lombardo M. The impact of vitamin D on immune function and its role in hashimoto’s thyroiditis: A narrative review. Life (Basel). (2024) 14:771. doi: 10.3390/life14060771, PMID: 38929753 PMC11204671

[70] Simsek Y Cakır I Yetmis M Dizdar OS Baspinar O Gokay F. Effects of Vitamin D treatment on thyroid autoimmunity. J Res Med Sci. (2016) 21:85. doi: 10.4103/1735-1995.192501, PMID: 28163731 PMC5244647

[71] Zhang J Chen Y Li H Li H. Effects of vitamin D on thyroid autoimmunity markers in Hashimoto’s thyroiditis: systematic review and meta-analysis. J Int Med Res. (2021) 49:3000605211060675. doi: 10.1177/03000605211060675, PMID: 34871506 PMC8711703

[72] Chahardoli R Saboor-Yaraghi AA Amouzegar A Khalili D Vakili AZ Azizi F. Can supplementation with vitamin D modify thyroid autoantibodies (Anti-TPO ab, anti-tg ab) and thyroid profile (T3, T4, TSH) in hashimoto’s thyroiditis? A double blind, randomized clinical trial. Horm Metab Res. (2019) 51:296–301. doi: 10.1055/a-0856-1044, PMID: 31071734

[73] Luo J Zhou L Sun A Yang H Zhang P Liu K. Herbal medicine for Hashimoto’s thyroiditis: A systematic review and network meta-analysis. J Ethnopharmacol. (2024) 323:117663. doi: 10.1016/j.jep.2023.117663, PMID: 38181936

[74] Liu H Tian Q Ai X Qin Y Cui Z Li M. Dihydroartemisinin attenuates autoimmune thyroiditis by inhibiting the CXCR3/PI3K/AKT/NF-κB signaling pathway. Oncotarget. (2017) 8:115028–40. doi: 10.18632/oncotarget.22854, PMID: 29383139 PMC5777751

[75] Zhou Y Shen H Lan W Shi Y Yao Q Wen W. Mechanism of Xiaoying Daotan decoction in treating Hashimoto’s thyroiditis based on the Notch/Treg/Th17 pathway. Ann Trans Med. (2021) 9:1760–0. doi: 10.21037/atm-21-6253, PMID: 35071454 PMC8756240

[76] Ma BE Chen D Liu Y Zhao Z Wang J Zhou G. Yanghe decoction suppresses the experimental autoimmune thyroiditis in rats by improving NLRP3 inflammasome and immune dysregulation. Front Pharmacol. (2021) 12. doi: 10.3389/fphar.2021.645354, PMID: 34234669 PMC8255388

[77] Guo Q Qu H Zhang H Zhong X. Prunella vulgaris L. Attenuates experimental autoimmune thyroiditis by inhibiting HMGB1/TLR9 signaling. Drug Des Devel Ther. (2021) 15:4559–74. doi: 10.2147/DDDT.S325814, PMID: 34764638 PMC8576104

[78] Aktaş T Celik SK Genc GC Arpaci D Can M Dursun A. Higher levels of serum TLR2 and TLR4 in patients with hashimoto’s thyroiditis. Endocrine Metab Immune Disord - Drug Targets. (2020) 20:118–26. doi: 10.2174/1871530319666190329114621, PMID: 30924423

[79] Zhao Z Li J Song N Gao H Jin Z Chen Y. Buzhong Yiqi decoction improves inflammation and oxidative damage in autoimmune thyroiditis by inhibiting apoptosis via the SIRT1-Mediated Nrf2/NF-κB axis. J Ethnopharmacology. (2025) 351:119967. doi: 10.1016/j.jep.2025.119967, PMID: 40360040

[80] Huang S Ziros PG Chartoumpekis DV Psarias G Duntas L Zuo X. Traditional chinese medicine for hashimoto’s thyroiditis: focus on selenium and antioxidant phytochemicals. Antioxidants (Basel). (2024) 13:868. doi: 10.3390/antiox13070868, PMID: 39061936 PMC11274136

[81] Zhu X Zhang C Feng S He R Zhang S. Intestinal microbiota regulates the gut-thyroid axis: the new dawn of improving Hashimoto thyroiditis. Clin Exp Med. (2024) 24:39. doi: 10.1007/s10238-024-01304-4, PMID: 38386169 PMC10884059

[82] Zhou L Luo JL Sun A Yang HY Lin YQ Han L. Clinical efficacy and molecular mechanism of Chinese medicine in the treatment of autoimmune thyroiditis. J Ethnopharmacol. (2024) 323:117689. doi: 10.1016/j.jep.2023.117689, PMID: 38160869

[83] Wang X Li Y Xie H Dai Z Ma L Zhu X. Effect of acupuncture on Hashimoto thyroiditis: A systematic review and meta-analysis. Med (Baltimore). (2024) 103:e37326. doi: 10.1097/MD.0000000000037326, PMID: 38428856 PMC10906624

[84] Cheng F-K. An overview of the contribution of acupuncture to thyroid disorders. J Integr Med. (2018) 16:375–83. doi: 10.1016/j.joim.2018.09.002, PMID: 30341025

[85] Wang S Yang C Zeng W Tian H Du S Zhao J. Acupuncture treatment for Hashimoto’s thyroiditis: An exploratory randomized controlled trial. Integr Med Res. (2024) 13:101023. doi: 10.1016/j.imr.2024.101023, PMID: 38420579 PMC10899034

[86] Serrao-Brown H Saadi A Wong J Papachristos A Sywak M Sidhu S. Outcomes of thyroidectomy in symptomatic, euthyroid Hashimoto’s patients: a case control study. ANZ J Surg. (2024) 94:1800–5. doi: 10.1111/ans.19155, PMID: 39011996

[87] Thatipamala P Noel JE Orloff L. Quality of life after thyroidectomy for hashimoto disease in patients with persistent symptoms. Ear Nose Throat J. (2022) 101:NP299–304. doi: 10.1177/0145561320967332, PMID: 33090901

[88] Zivaljevic VR Bacotic BRB Sipetic SB Stanisavljevic DM Maksimovic JM Diklic AD. Quality of life improvement in patients with Hashimoto thyroiditis and other goiters after surgery: A prospective cohort study. Int J Surg. (2015) 21:150–5. doi: 10.1016/j.ijsu.2015.08.001, PMID: 26254997

